# Hidden middle governance reduces antibiotic residue health risks and food loss in aquatic supply chains

**DOI:** 10.1016/j.isci.2026.115097

**Published:** 2026-02-20

**Authors:** Cangyu Jin, Zhengcong Wang, Weihua Zhou, Siwei Fu

**Affiliations:** 1 School of Management, Zhejiang University, Hangzhou 310058, China; 2 College of Economics and Management, Huazhong Agricultural University, Wuhan 430070, China

**Keywords:** Aquatic science, Aquatic biology, Food science, Food microbiology, Aquaculture, Aquaculture diseases

## Abstract

The “hidden middle” of food supply chains, referring to the small- and medium-scale intermediaries linking producers to markets, often obscures food safety risks and exacerbates losses. Integrating aquatic product testing data from 247 Chinese prefectures (2015–2022), we assessed the cross-regional impact of antibiotic residues (ARs) through the hidden middle of China’s aquatic supply chains. We found that AR above legal limits caused 44,200 tons of food loss and health risks exceeding safe thresholds in six provinces. We then developed an optimization model to allocate sampling resources, resulting in 14,702 tons of food loss reduction and 77% decrease in health risks. Relative to current monitoring schemes, the optimized strategy delivered an additional 6,951-ton reduction in food loss and a 17% larger risk reduction per province per year. Our results demonstrate that focusing governance on AR spread through the hidden middle can improve food safety and reduce risks to food security and public health.

## Highlights

• Data-driven mapping identifies antibiotic residue risk in aquatic supply chains
• The “hidden middle” obscures antibiotic risks in aquatic supply chains
• Residue-related health risks affect consumers in both coastal and inland provinces
• Optimized governance efficiently reduces food loss and lowers health risks

## Graphical abstract

**Figure:**
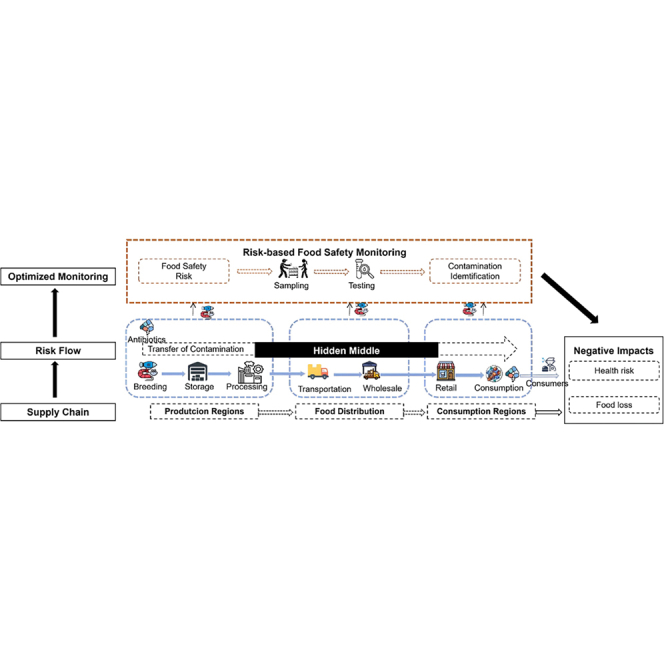

## Introduction

The misuse and overuse of veterinary antibiotics can lead to antibiotic residues in food products and antimicrobial resistance (AMR) in food systems, further posing significant threats to public health and food security.^1^^,^^2^^,^^3^^,^^4^^,^^5^ Especially aquatic products, the global yield of aquatic products has rapidly increased, surpassing that of beef over the past 20 years.^6^^,^^7^ While the high protein and low-fat characteristics of aquatic products meet the growing health demands and preferences,^7^^,^^8^ the supply chain facilitates the distribution of locally developed bacteria on a global scale, serving as a potential pathway for transmitting antibiotic residue (AR), AMR bacteria (AMRB), and AMR genes (AMRGs) from farming to humans.^9^^,^^10^^,^^11^ Furthermore, compared to other animal-derived foods, aquatic products are more likely to be consumed raw,^7^^,^^12^ increasing the risk of AR and related risks transmission.^11^

China, as the largest producer, exporter, and consumer of aquatic products, acts as the central hub in the global aquatic product supply chain.^13^ However, a distinct structural disconnect exists between production and consumption. On the production side, millions of small-scale farmers operate with low modernization levels, exacerbating antibiotic misuse.^14^^,^^15^ On the distribution side, over 70% of aquaculture products in China are channeled into the consumer market through a distribution network dominated by farmers, brokers, wholesale markets, and wet markets, which serve as the primary retail outlets.^16^

### The hidden middle of the food supply chain

**Figure 1:**
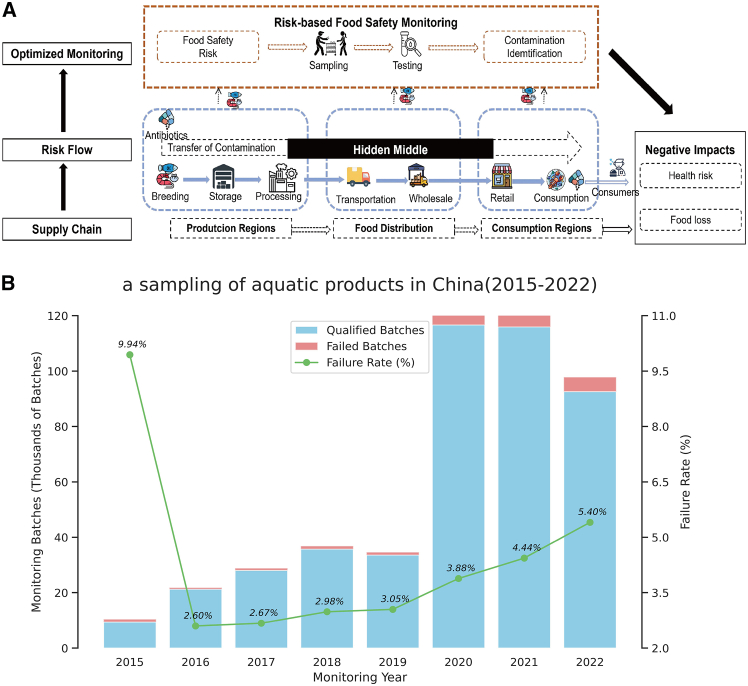
The food safety monitoring scheme with a hidden middle of the aquatic supply chain and the current contamination of aquatic products in China (A) The framework of linking a hidden middle of the aquatic supply chain and respective food safety monitoring schemes. (B) Sampling of aquatic products in China (2015–2022).

We conceptualize the widespread presence of small- and medium-scale actors as the “hidden middle” (Figure 1A),^7^^,^^17^^,^^18^ a critical blind spot in food safety governance. Theoretically, the “hidden middle” is characterized by high fragmentation and informality, which creates severe information asymmetry between producers and regulators. In the context of supply chain traceability, these actors form a “black box” that severs the information link, preventing effective accountability. This opacity allows high-risk products to blend with compliant ones, a classic “lemon market” problem, facilitating “free-riding” behaviors where safety standards are ignored.^19^ The structural vulnerability was starkly illustrated by the July 2024 “Cooking Oil in Dirty Fuel Tankers” scandal in China, which exposed how untraceable logistics can undermine even the strictest food safety laws. Furthermore, the rivalry among regional governments under the tax-sharing system has impeded cross-regional governance, obstructing the management of residue transmission across production and consumption areas.^7^^,^^20^

Despite the urgency of this issue, existing literature presents distinct gaps in quantifying these risks. First, focus on the static assessment of localized contamination while overlooking the dynamic transmission process. Regarding the assessment of AR, previous studies have extensively mapped the regional distribution of AR,^21^^,^^22^ environmental resistance genes,^23^^,^^24^^,^^25^ and environmental and ecological health assessments of AR.^26^^,^^27^^,^^28^^,^^29^^,^^30^ However, these works often treat risks as static points rather than dynamic flows. Although these works establish a critical data foundation, they largely treat contamination as discrete, static incidents, failing to account for the geographic separation between aquatic supply and demand. Drawing on the concept of “telecoupling,”^31^ it becomes evident that socioeconomic and environmental impacts act across vast distances. In such a system, risk is not stationary; it possesses transboundary transmissibility. Thus, traditional static monitoring is ill-equipped to assess the dynamic health risks embedded within and driven by globalized supply chain networks.^32^^,^^33^^,^^34^^,^^35^

Second, concerning risk governance, the prevailing literature predominantly emphasizes technological interventions,^36^^,^^37^^,^^38^^,^^39^ or the optimization of government regulatory efficiency.^40^^,^^41^^,^^42^ However, these paradigms implicitly presuppose a transparent, vertically integrated supply chain structure. They largely fail to account for the “hidden middle,” the fragmented network of informal intermediaries that dominates the aquatic sector.^17^^,^^43^ This structural opacity creates severe information asymmetry between upstream producers and downstream regulators. As elucidated by operations management theory, such low visibility diminishes the effectiveness of external monitoring^44^ and creates structural incentives for economically motivated adulteration.^19^ Furthermore, in the absence of credible traceability, supply chain actors are prone to moral hazard, engaging in “free-riding” behaviors where safety standards are compromised to reduce costs.^45^^,^^46^ Consequently, standard high-tech solutions are often rendered ineffective in these non-integrated, opaque market environments.

Third, while the analytical framework of embodied flows has notably evolved from tracking virtual resources to monitoring hazardous chemicals and their associated health burdens in trade, a critical methodological gap remains in quantifying the cross-regional transmission of foodborne safety risks. Existing literature has established the global food system as a primary transport pathway for contaminants, effectively displacing production-side health risks away from the beneficiaries of consumption.^47^^,^^48^ Specifically, recent studies have characterized the embodied flows of hazardous chemical pollutants, such as per- and polyfluoroalkyl substances (PFASs),^49^ polychlorinated biphenyls (PCBs),^50^ and mycotoxins,^51^ demonstrating how inter-regional trade redistributes exposure risks and drives distant health damages, including air quality-related mortality.^52^^,^^53^ Qiu et al.,^49^ for instance, explicitly quantified the global amplification of PFAS exposure through marine fish trade, highlighting the pivotal role of trade networks in contaminant diffusion. However, despite these advances in mapping stable environmental pollutants, the embodied risk of antibiotic residues remains insufficiently explored. Unlike the contaminants modeled in previous studies, antibiotic residues involve complex transmission dynamics masked by the structural opacity of non-traceable intermediaries, especially in China. Consequently, a data-driven approach that conceptualizes antibiotic residues as embodied food safety risks, which flow through and are amplified by untraceable intermediaries, remains underexplored. By adapting this interdisciplinary approach, the present study transcends static, localized detection and offers a systemic quantification of risk transferring across complex supply chains.

### Data-driven food safety monitoring

It is worth noting that data-driven food safety monitoring provides a big data foundation for controlling the cross-regional AR risk flow in the aquatic supply chain and offers insights into managing the potential risk of public health and food security.^18^^,^^38^ Strengthening surveillance to guide AR poses a significant challenge across sectors, particularly in the aquatic supply chain and fisheries industry.^11^^,^^54^ Currently, even for the most ubiquitous aquatic taxa consumed worldwide, such as freshwater and marine fish, epidemiological data remain fragmented, with a distinct absence of integrated, systematic surveillance mechanisms targeting foodborne pathogens. The primary means of antibiotic risk governance are hampered by governance costs and insufficient regulatory resource allocation.^55^^,^^56^ Data-driven food safety monitoring is a crucial governmental tool that addresses various food safety risks, including AR in the aquatic market. The advancement in data-driven technology provides an effective means of managing AR health risks, particularly within complex agricultural product supply chains.^18^^,^^57^

### Study objectives and contribution

While studies have addressed general supply chain traceability and local antibiotic contamination, few have quantified the cross-regional transmission of AR risk specifically through the lens of untraceable intermediaries. The link between the structural opacity of the “hidden middle” and the spatial diffusion of health risks remains under-explored. When regulatory enforcement is effective, but sampling resources are limited (Figure 1B), identifying these transmission pathways becomes critical for alleviating the “barrel effect” in food safety governance. To bridge this gap, this study integrates the “hidden middle” concept with antibiotic transmission dynamics. The objective of this study is to quantify the food loss and health risks associated with AR and to develop an optimized monitoring strategy that accounts for the supply chain’s hidden structures.

**Figure 2:**
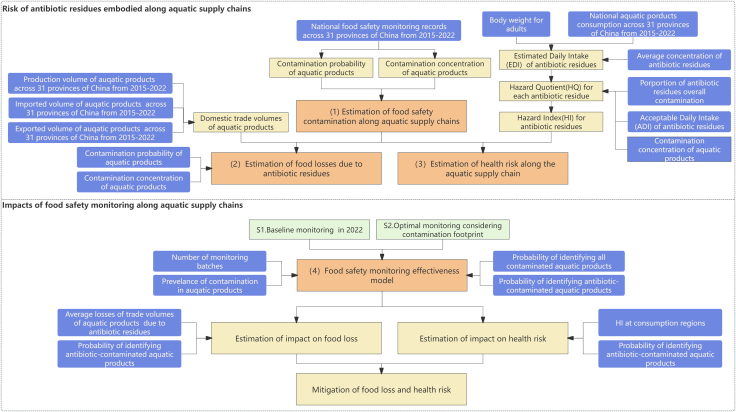
The framework of methodology The orange boxes represent workflow processes; the blue boxes represent input data in the model; the yellow boxes represent output; the green boxes represent the food safety monitoring scenarios.

This study contributes to the literature in two ways. On one hand, it extends the “embodied flow” framework to food safety, providing a theoretical argument for why the “hidden middle” creates critical information asymmetries that disrupt risk governance (Figure 2). On the other hand, it proposes a spatially optimized monitoring strategy. By integrating data-driven supply chain analytics, we compare the effects of our optimized approach against current protocols, offering a cost-effective solution to reduce antibiotic-related food loss and protect public health.

## Results

### Antibiotic contamination across regions in China

**Figure 3:**
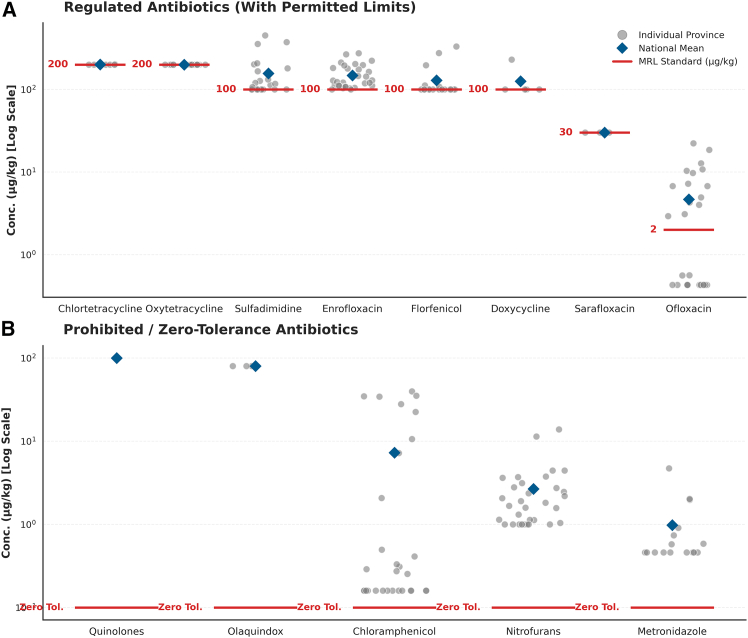
Prevalence of antibiotic residues in aquatic products across Chinese provinces (2015–2022) (A) Distribution of regulated antibiotics with specific maximum residue limits (MRLs). Red lines indicate the permissible MRL values (μg/kg) for each antibiotic. (B) Distribution of prohibited or zero-tolerance antibiotics. Red lines mark the zero tolerance threshold (plotted at 0.1 μg/kg for visualization on the logarithmic scale). Notes: In both panels, gray dots represent the mean concentration detected in individual provinces, illustrating the widespread nature of contamination. Blue diamonds indicate the national mean concentration for each antibiotic. The y axis is presented on a logarithmic scale to accommodate the wide range of concentration values.

Analysis of AR in aquatic products across 31 Chinese provinces (2015–2022) reveals widespread contamination exceeding regulatory standards (see Figure 3; Figure S4). Despite the implementation of a zero-tolerance policy, prohibited antibiotics were persistently detected. Notably, although Olaquindox contamination was localized to Jiangsu, Qinghai, and Sichuan, it represented the most severe violation with a mean concentration of 80.00 μg/kg, followed by Chloramphenicol (7.29 μg/kg) and Nitrofurans. Similarly, regulated antibiotics frequently surpassed their respective maximum residue limits (MRLs), challenging the assumption of general compliance. Specifically, Ofloxacin exhibited a mean concentration of 4.67 μg/kg (MRL 2 μg/kg), while Sulfadimidine (156.47 μg/kg), Enrofloxacin (147.85 μg/kg), Florfenicol, and Doxycycline all averaged above the 100 μg/kg limit. Even for antibiotics with higher thresholds, such as Chlortetracycline and Oxytetracycline, the mean concentrations aligned precisely with the MRL threshold of 200 μg/kg. These distribution patterns, which align with regional consumption trends (see Figure S1), highlight significant supply chain risks and establish the basis for our subsequent exposure assessments (EDI and HQ).

### The role of the hidden middle in antibiotic residue transmission

**Figure 4:**
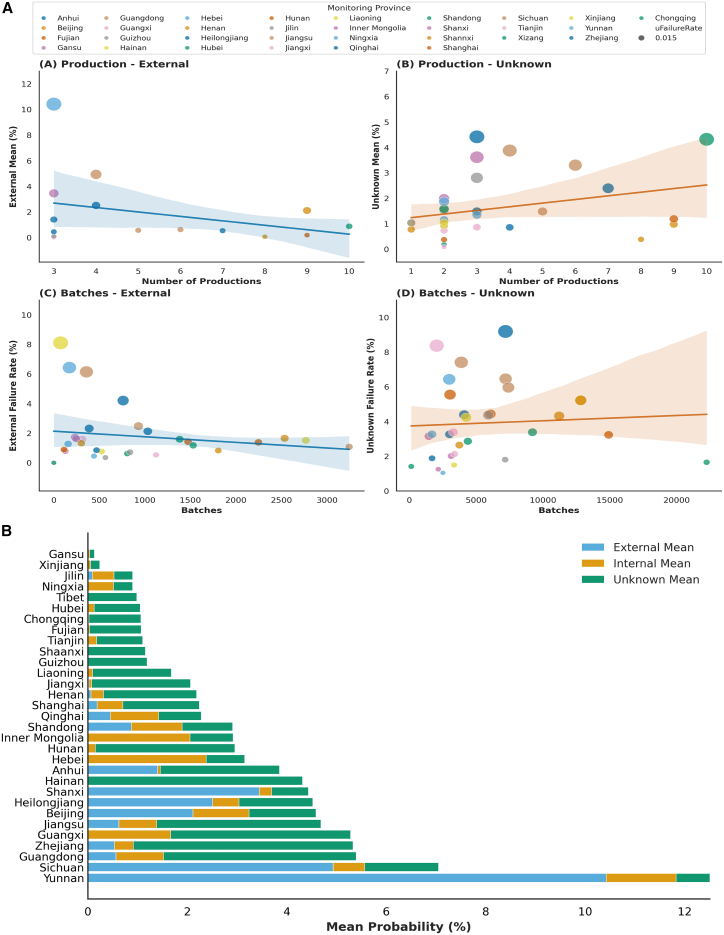
The probability of aquatic products contaminated with antibiotic residues transferring from production regions to consumption regions in China during 2015 and 2022 (A) The relationship between the number of production provinces and the probability of externally sourced and unknown-sourced antibiotic residues in aquatic products. The position of each dot represents the external-sourced/unknown-sourced antibiotic residues the probability of one province. The error bands (95% confidence) show the best fit for linear regression by ordinary least squares. The fitted curve on the left side, representing the relationship between the number of production provinces and the probability of external-sourced antibiotic residues in aquatic products, has a slope of -0.0035 ± 0.05 (95%CI, R^2^ = 0.1078), and the intercept term is 0.0373. The fitted curve on the right side, representing the relationship between the number of production provinces and the probability of unknown-sourced antibiotic residues in aquatic products, has a slope of 0.0014 ± 0.05(95%CI, R^2^ = 0.929), and the intercept term is 0.011. The uFailure dot with value of 0.015 is used as a benchmark. (B) Antibiotic residue flows in the aquatic supply chain in China.

Based on aquatic products sampling cases from 2015 to 2022, we explored the role of the “hidden middle” in the regional transfer of AR risk (Figure 4). Sources were categorized into three distinct streams, namely internal (local origin), external (*trans*-provincial), and unknown (untraceable products characterizing the “hidden middle”). Figure 4A reveals divergent correlation patterns. For traceable external sources, we observed a negative linear relationship between source diversity and risk probability, with risk decreasing by 0.35% for each additional provenance province. Conversely, unknown origins exhibited a positive linear correlation, where risk increased by 0.14% per additional unit of source complexity. Notably, the non-compliance rate of unknown sources remained unresponsive to increased monitoring frequency. This positive correlation suggests a critical behavioral mechanism driven by strategic obfuscation and adverse selection. In markets plagued by information asymmetry, high-risk actors are incentivized to exploit fragmented “hidden middle” to conceal non-compliance and evade regulatory accountability.^19^ Unlike traceable external sources, which typically originate from regulated, large-scale production bases and thus show a negative correlation with risk, unknown sources represent an accountability vacuum. This opacity creates a haven for moral hazard, allowing high-risk actors to “free-ride” on the general market reputation while minimizing the risk of detection. Therefore, a higher proportion of unknown origins does not merely reflect logistical complexity; it serves as a distinct risk signal, indicating a deliberate decoupling of product flow from information flow to mask food safety failures. Figure 4B quantifies these disparities. Average contamination probabilities were 0.60%(internal), 1.88%(external), and 1.60%(unknown). These comparisons underscore that traceability serves as a proxy for regulatory oversight, increased diversity of regulated sources correlates with reduced risk, whereas the expansion of untraceable “hidden middle” channels is associated with amplified contamination risk.

### Food loss and health risks

**Figure 5:**
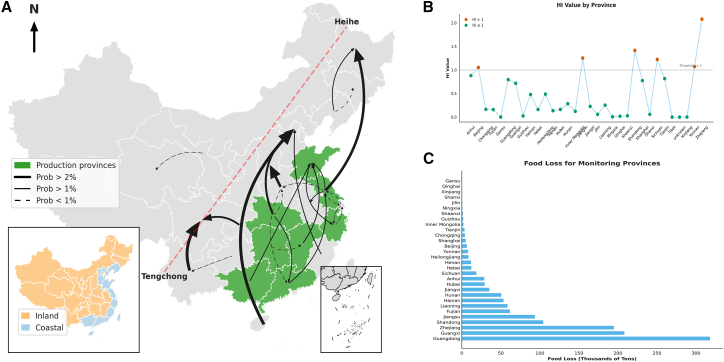
The spatial distribution of antibiotic residue, along with the food loss and Hazard Index (HI) caused by it in aquatic products (2015–2022) (A) Comparison across Chinese provinces of antibiotic residue contamination probability (%) averaged over internal-sourced, external-sourced, and unknown-sourced during 2015 and 2022. (B) The aquatic product loss between regions in China due to antibiotic residues. (C) Antibiotic residue HI values across Chinese provinces, and the HI represents the cumulative non-carcinogenic health risk from dietary exposure to multiple antibiotics, with an HI value greater than 1 indicating potential health concern.

Figure 5A illustrates the pronounced spatial heterogeneity in the probability of AR originating from external sources. We identified three distinct patterns of cross-regional risk transmission. First, a coastal-to-inland gradient; second, a dominant flow from major production provinces to consumption provinces; and third, a high concentration of risk in densely populated regions east of the Hu Huanyong Line. Food loss and health risks (quantified by Hazard Index, HI) were estimated by integrating contamination prevalence with market circulation volumes (Figure 5). Figure 5B depicts the aggregate food loss across regions in China from 2015 to 2022. High-loss regions are predominantly clustered in major aquatic product-producing provinces, specifically Guangdong, Guangxi, and the eastern coastal provinces. The annual average food loss per province is 44,200 tons, with Guangdong having the highest loss volume. Figure 5C evaluates the provincial level HI of multiple AR in aquatic products in China, adhering to the risk assessment framework initially established by the USEPA (U.S. Environmental Protection Agency) and later adopted by the WHO. During 2015–2022, six provinces recorded HI values for AR exceeding the critical threshold of 1, indicating that the cumulative exposure to AR in these regions may pose significant health risks. Coastal provinces such as Jiangsu, Shandong, and Zhejiang exhibited particularly elevated HI values, reflecting the direct impact of intensive aquaculture, underscoring the heightened health risks associated with AR in the aquatic product supply chain. Furthermore, inland provinces including Beijing, Sichuan, and Yunnan also registered HI values above 1, suggesting that despite not being primary production areas, these regions remained vulnerable to considerable health risks driven by cross-regional supply chain interactions.

### Optimized monitoring strategies and antibiotic residue risk mitigation

**Figure 6:**
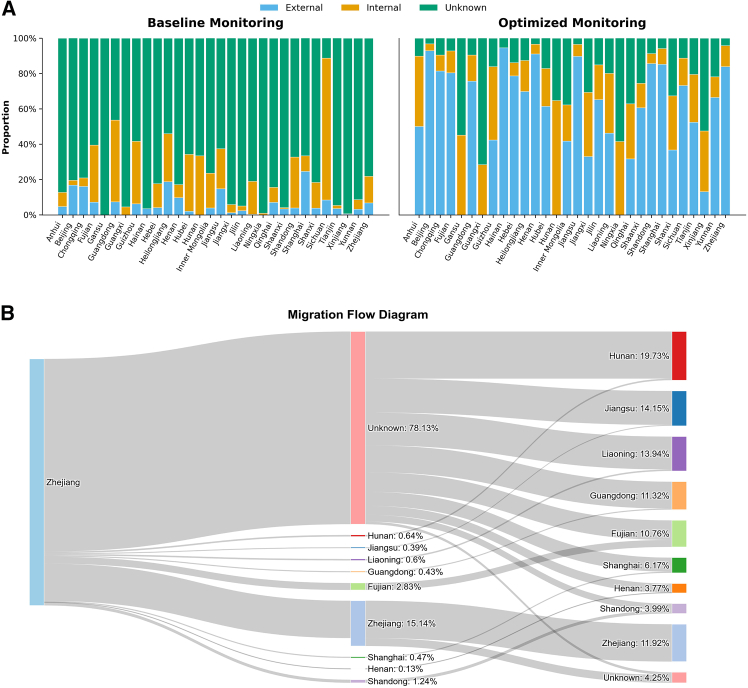
Comparison of aquatic product monitoring batches in China before and after optimization (A) The actual and optimized monitoring batches of aquatic products from internal sources, external sources, and unknown sources in China in 2022. A 100% stacked bar chart represents the proportions of internal sources, external sources, and unknown sources, with the total summing to 100%. (B) The optimized flow of inspection batches from different source provinces in an aquatic product monitoring province in 2022, exemplified by Zhejiang province. Node, in the Sankey diagram, nodes represent the monitoring and production provinces of the inspected samples, specifically indicating which provinces provide the samples monitored by Zhejiang provinces producing these aquatic products. Each flow, connecting the nodes, represents the movement of aquatic products from the producing provinces to Zhejiang. The width of the flow indicates the number of batches; wider flows represent a greater number of batches, while narrower flows indicate fewer batches. Color, different colors represent different provinces or distinct inspection statuses (actual monitoring batches in 2022 and optimized monitoring batches in 2022); this allows for a clear visualization of how inspection batches vary across different origins and inspection states. Comparison of actual monitoring batches in 2022 and optimized monitoring batches, by comparing the “actual monitoring batches in 2022” with “optimized monitoring batches,” one can observe how the optimized batches are allocated between the monitoring province (Zhejiang) and other provinces.

Our findings indicated the transfer of AR within China’s aquatic product supply chain from production provinces to consumption provinces (monitoring provinces), leading to food losses and potentially posing health risks to residents in certain provinces. To further explore methods for controlling AR in aquatic products, we developed an optimization model to simulate the maximization of the probability of identifying contaminated aquatic products from all production sources at each province in China, constrained by fixed sampling resources (Figure 6 and STAR Methods). We used the most recent 2022 China aquatic product monitoring program as the baseline. The model quantitatively evaluated and compared the effectiveness of the optimal food safety monitoring scheme with the baseline monitoring scheme across different consumption provinces, in terms of sampling resource efficiency, the reduction of food loss due to AR, and the mitigation of health risk due to antibiotic residues. Under the current system, China’s aquatic product monitoring plan targets all types of contaminants. Therefore, in the model for evaluating regulatory effectiveness, our goal was to maximize the detection of aquatic products that pose a food safety risk (including not only antibiotic residues but also other contaminants), within the constraint of fixed monitoring resources across all sources.

We simulated the distribution of optimal monitoring batches and compared the baseline monitoring batches with the optimized distribution (Figure 6A). The probabilities of identifying aquatic products contaminated with any contaminant were improved at each consumption province after optimizing the monitoring schemes (Figure S7). The probabilities of identifying aquatic products contaminated with AR were also improved at most consumption provinces (26 provinces) after optimizing the monitoring schemes (Figure S8). The results showed that the proportion of monitoring from unknown sources was significantly reduced in each province after optimization, while the proportion of external aquatic product monitoring increased. To further understand the origin and quantity distribution of aquatic product monitoring batches for the regulatory provinces in terms of the baseline monitoring scheme and the optimal monitoring scheme, we used Zhejiang (China’s leading province for both aquatic product production and consumption) as an example. By observing the changes in sampling batches before and after optimization, we aimed to assess whether the allocation of the optimized monitoring batches was more rational or balanced (Figure 6B). Our analysis reveals that sampling batches in Zhejiang significantly reduced the proportion of monitoring from unknown sources, from 78.13% to 4.25%, while also decreasing the internal monitoring proportion from 15.14% to 10.76%. Concurrently, the external monitoring proportion was markedly increased from 6.73% to 83.83%.

### Effectiveness of optimal monitoring strategies

**Figure 7:**
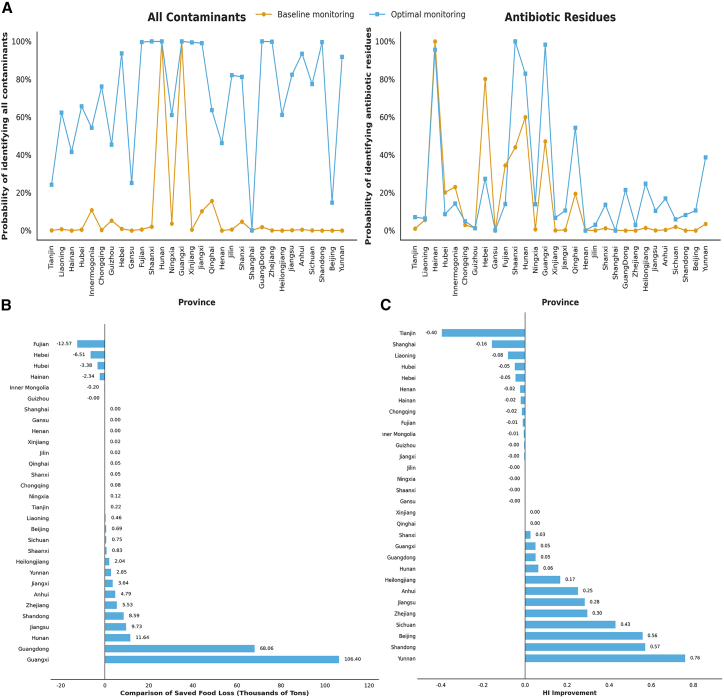
Impacts of optimal monitoring schemes on food loss and HI (Hazard Index) (2015–2022) (A) Effectiveness of the optimized monitoring strategy in detecting all contaminants and antibiotic residues across consumption provinces. Effectiveness is expressed as the probability of identifying (i) any contaminant and (ii) antibiotic residues specifically, among products originating from all production sources. (B) Reduced food loss due to antibiotic residues for province after implementing optimal monitoring compared with baseline monitoring. (C) Improvement in HI achieved by the optimized monitoring strategy compared with the baseline. Positive HI improvement values (>0) indicate greater reductions in cumulative health risk under the optimized monitoring scheme relative to the baseline.

We estimate the comparative efficacy of baseline versus optimized monitoring strategies in detecting general contaminants and specific AR (Figure 7A). The optimized protocol (red line) consistently outperformed the baseline approach (blue line) across diverse consumption provinces, exhibiting substantial improvements in detection rates for both general contaminants and AR. These results underscore the significant regional variability in regulatory efficiency and demonstrate the superior detection capabilities achieved through optimization. Figures 7B and 7C quantify the tangible benefits of this strategic shift in terms of food loss reduction and health risk mitigation. Figure 7B reveals significant regional variation in the effectiveness of optimal food safety monitoring in reducing food loss associated with AR in aquatic products compared with baseline monitoring scheme (the results of impacts on food loss caused by all contaminants in Figures S9 and S10). We finally compared the reduction in health risk between optimized monitoring and baseline monitoring (Figure 7C). By recalibrating resource allocation to specifically target risks emanating from the “hidden middle,” the optimized strategy achieved a projected mitigation of 14,702 tons of food loss and a 77% reduction in cumulative health risks within the national aquatic supply chain. A net improvement of 6,951 tons of food loss and 17% health risk reduction compared to the baseline monitoring approach (Figures S9–S11). The food loss due to AR in various provinces of China saved by baseline monitoring in 2022 (Figure S9) and by optimal monitoring (Figure S10). Additionally, the left health risks associated with AR across China’s provinces after implementing the baseline monitoring (Figure S11) and the optimal monitoring (Figure S12) are provided in Supplementary Materials.

### Sensitivity analysis

The robustness of our core findings was corroborated through comprehensive sensitivity analyses (Figures S1 and S2). We tested the model’s stability against perturbations in key parameters, including contamination rates and average residue concentrations. First, the stability of health risk estimates. The spatial identification of high-risk provinces (HI > 1) displayed high stability. When both contamination rates were increased by +10% and mean concentrations by +20%, four additional provinces, Shanghai, Guangdong, Tianjin, and Anhui, exceeded the HI > 1 threshold. Conversely, when contamination rates and concentrations were decreased by −10% and −20%, respectively, only Zhejiang and Shandong remained above the health-risk threshold. Despite these numerical shifts, the overall spatial pattern and relative health-risk ranking were highly consistent across all scenarios. In every sensitivity condition, the top six provinces with the greatest health risks remained the same as in the baseline estimation, namely Zhejiang, Shandong, Jiangsu, Sichuan, Yunnan, and Beijing. Second, the stability of food loss estimates. Food loss estimates were recalculated under the adjusted contamination-rate scenarios. Across the majority of provinces, the magnitude of food loss attributable to AR exhibited high stability, with only Guangdong showing a marginal divergence between scenarios. Despite these minor numerical differences, the overall spatial pattern and relative ranking of provincial food loss remained congruent with the baseline results, confirming the reliability of the estimation framework. As to the optimization strategy, the relative effectiveness of the optimized monitoring strategy compared with the conventional scheme remained robust across all sensitivity scenarios. The performance advantage of the optimized strategy persisted for nearly all provinces, with only Yunnan, Shandong, and Beijing exhibiting negligible fluctuations in the magnitude of health-risk reduction. Similarly, the estimated food loss saved under the optimized strategy maintained patterns consistent with baseline estimations. Overall, the sensitivity analyses demonstrate that the core quantitative conclusions, particularly the spatial distribution of health risk and the superiority of the optimized monitoring strategy, are robust to uncertainty in the key model parameters.

### Comparison with previous frameworks

Our exposure and health-risk estimations align with internationally established protocols for veterinary drug residue assessment, including those developed by Codex Alimentarius, FAO, WHO, and JECFA, which recommend comparing EDI with ADI and summarizing results using hazard quotients and hazard indices.^58^^,^^59^^,^^60^ Previous studies examining AR in Chinese aquaculture have generally reported low to moderate risks when evaluating single antibiotics, but repeatedly identify fluoroquinolones and chloramphenicol-related compounds as major contributors to elevated exposure.^61^^,^^62^^,^^63^^,^^64^ Our findings are consistent with this literature: coastal, high-production provinces (e.g., Jiangsu, Shandong, and Zhejiang) show higher contamination and risk, and our cumulative HI estimates expand earlier assessments by identifying inland consumption centers (such as Beijing, Sichuan, and Yunnan) where multi-residue exposure exceeds health risk benchmark due to cross-regional trade flows. Global mapping initiatives for antimicrobial use and AMR similarly identify aquaculture production zones as critical hotspots for antibiotic inputs and potential human exposure via food systems.^4^^,^^11^ These international assessments emphasize the role of supply chain pathways, limited traceability, and mid-chain actors in shaping foodborne AMR and chemical risks, patterns that are reflected in our finding that “hidden middle” flows significantly contribute to both food loss and health risks. Our results, therefore, reinforce conclusions from global frameworks while extending them by quantifying how contamination, food loss, and cumulative health risks propagate along domestic aquatic supply chains, and by demonstrating that optimized monitoring strategies can more accurately target high-risk provinces influenced by these flows.

## Discussion

Our results indicated that the “hidden middle” in China’s aquatic product supply chain contributed to increased food loss and health risk caused by potential AR contamination, particularly in markets with diversified sources (internal, external, and unknown) of aquatic products. These risks propagated from production to consumption regions. This discovery underscores the critical importance of data-driven food safety risk management approaches in addressing the “hidden middle,” as traditional research methods struggle to fully utilize the vast amount of untraceable sampling data in food safety tests, which tend to prioritize traceability over actual food safety risks. Quantifying how the “hidden middle” connects production and consumption is essential for mapping risk-transmission pathways, estimating contamination-induced losses, and targeting regulatory resources to the highest-risk nodes. In turn, clarifying how these mid-chain processes elevate antibiotic residue-related health risks can inform strategies to reduce residue exposure and mitigate the downstream threat of antibiotic resistance. In this section, we will first discuss the estimation of how the “hidden middle” in the food supply chain exacerbated food loss and health risks caused by potential AR contamination, followed by an exploration of how optimizing regulatory approaches can address the role of the “hidden middle” in the supply chain (As shown in Figure 1A).

To illustrate how the “hidden middle” in the food supply chain exacerbates food loss and the health risks associated with potential AR contamination, we conducted multilevel risk analyses based on conditional probability. We first examined the extent to which the “hidden middle” in the aquatic supply chain contributed to the transfer of AR risks from production to consumption. Widespread contamination by AR exceeding MRLs was observed across China, with a clear shift of contamination from the production areas to the consumption regions. The result was consistent with a previous study, which found that the distribution of the AMR burden among urban populations in China is similar, with the majority concentrated in the region to the east of the “Hu huanyong Line.”^65^ This further confirmed AMR correlated with these residues.^2^^,^^66^ We also found that when the origin of aquatic products was unknown, the mean AR risk increased substantially. This was associated with considerable food loss at the provincial level and HI values exceeding 1 in six non-production provinces, including Beijing, Sichuan, and Yunnan. This pattern reflects the redistribution of contamination risks through interprovincial trade and cold-chain logistics, as aquatic products from coastal regions with intensive antibiotic use are widely supplied to inland markets. Such redistribution indicates that health risks are propagated through supply chain networks rather than restricted to production zones, emphasizing the need to extend monitoring and risk mitigation efforts to external production sources, as reflected by our optimal monitoring results.^10^^,^^67^ These findings emphasize the urgent need for stringent monitoring and regulation of antibiotic use in aquaculture to mitigate health risks and economic losses.^54^ Improving supply chain transparency and traceability remains essential for identifying contamination sources and preventing the spread of residues,^68^ thereby enhancing food safety, protecting public health, and mitigating food losses associated with antibiotic contamination.

The optimal monitoring schemes underscore the crucial role of strategically targeted surveillance in safeguarding food safety and public health. Strengthening traceability across the supply chain is essential for identifying contamination sources and preventing the spread of AR, in line with FAO and WHO guidance that effective monitoring and surveillance systems are key to managing food safety risks, including those arising from AR in aquaculture products.^69^ It is important to note that the systematic monitoring of foodborne AR in China with regularized surveillance protocols commencing only as recently as 2018.^70^ Global AR and AMR monitoring primarily relies on point prevalence surveys, which fall short of the monitoring capacity needed for effective control.^5^ Regulatory agencies could leverage these data-driven surveillance approaches to mitigate AR throughout the aquatic product supply chain, offering a viable pathway for managing AR in related agricultural and food products. By reallocating sampling efforts toward external sources and reducing reliance on unknown-source sampling, authorities can more accurately assess and mitigate risks associated with antibiotic contamination, aligning with studies that emphasize the importance of efficient monitoring systems in reducing economic losses related to food safety incidents.^71^^,^^72^^,^^73^^,^^74^^,^^75^

Our results also demonstrate that reallocating monitoring efforts from aquatic products of unverifiable origin to those clearly traced to external provinces effectively mitigates the hidden middle’s amplification of food losses and contaminant-related health risks. The significant reduction in food losses underscores the economic benefits of optimizing monitoring distribution, as antibiotic contamination can lead to substantial economic losses due to rejected exports, product recalls, and decreased consumer confidence^74^^,^^76^^,^^77^; improving the allocation of monitoring efforts protects the aquaculture industry’s economic interests while ensuring product safety. Moreover, increasing external-source monitoring acknowledges the interconnected nature of regional supply chains and the need for comprehensive monitoring, improving early detection and intervention of AR, which can be introduced at multiple points, before products reach consumers.^77^ Reallocating monitoring resources according to identified risk flows marks a major step forward in managing antibiotic residues in China’s aquatic product supply chain. By improving traceability and strategically targeting surveillance efforts, this approach can significantly reduce health risks and economic losses, establishing optimized monitoring as a fundamental pillar of aquaculture food safety management.

Our findings are consistent with international assessments of veterinary drug residues and foodborne AMR. FAO/WHO and JECFA guidelines similarly evaluate exposure by comparing estimated daily intake with acceptable daily intake using HQ and HI metrics.^59^^,^^78^^,^^79^ The patterns observed in China—particularly the concentration of risks in major aquaculture regions and the amplification of exposure in high-consumption inland markets—mirror challenges reported in the European Union’s risk-based monitoring and official control systems under Regulation (EU) 2017/625, where targeted sampling and RASFF notifications frequently highlight residues and traceability gaps along complex supply chains.^80^^,^^81^ Similar issues have been documented in Southeast Asia: in Vietnam, monitoring studies and policy reviews describe the occurrence of AR in aquaculture and note limited enforcement capacity and fragmented surveillance systems,^10^^,^^82^^,^^83^ while in Thailand, recent work on small-scale freshwater aquaculture farms reports ongoing concerns around antimicrobial use and the need for stronger governance and support mechanisms.^84^^,^^85^ These international parallels reinforce our conclusion that supply-chain transparency and risk-based resource allocation are critical components of effective residue-control strategies, and they highlight the importance of strengthening monitoring frameworks to address the role of the “hidden middle” in transmitting antibiotic-related risks.

Based on our quantitative findings, we propose two primary mechanistic hypotheses to explain how the “hidden middle” facilitates the cross-regional transmission of AR/ARB/AMR/AMRG risks. First, logistics-facilitated diffusion. The rapid development of China’s cold-chain logistics network acts as a double-edged sword. While it effectively reduces physical food loss by extending shelf life, it simultaneously extends the lifespan and travel distance of these risks. Within the “hidden middle,” logistics are often managed by fragmented, third-party providers who lack the capability or incentive to verify safety compliance. This logistical opacity allows contaminated products to be rapidly distributed from local farms to distant national markets before risks can be detected, effectively bypassing local containment. Second, regulatory arbitrage and asymmetry. We hypothesize that regulatory asymmetry between regions drives the direction of risk flow. Production-concentrated provinces often prioritize yield and economic output, which may lead to looser enforcement of antibiotic restrictions. In contrast, consumption-concentrated metropolises enforce stricter market access standards but suffer from supply deficits. This imbalance creates an opportunity for regulatory arbitrage, where high-risk products originating from areas with lower enforcement costs flow into high-demand markets, exploiting the information gap inherent in cross-regional trade to evade inspection.

Future research should further test and refine these mechanisms by explicitly integrating data and drivers along the supply chain and in the broader socio-environmental context. One priority is to combine real-time logistics information (e.g., GPS-based tracking) with detailed transport- and cold-chain network data to map physical flow paths and quantify how spatial connectivity, trade volumes, and mid-chain actors shape cross-regional residue transmission, including potential regulatory arbitrage. In parallel, incorporating socioeconomic variables, such as market demand, production intensity, and regional regulatory and enforcement capacity, would help explain variation in antimicrobial use and contamination pathways across regions. Finally, integrating climate-related factors, including long-term variability and extreme weather events, could elucidate how environmental change alters aquaculture practices, disease pressures, and antibiotic application patterns, thereby creating new residue-transfer dynamics. Together, these extensions would enable more comprehensive modeling of emerging risks and support more adaptive, evidence-based monitoring and policy interventions.

### Limitations of the study

This study has several limitations related to data, methodology, and policy context. First, the sampling data used in our optimization model come from routine food safety inspections rather than a dedicated national antibiotic residue surveillance program; under the current Food Safety Law, inspection resources are allocated by food category, and specialized residue monitoring is conducted only sporadically by local authorities, which may underrepresent high-risk nodes. Second, our contamination and exposure assessments rely on aggregated monitoring results at the consumption-region level because origin-specific concentration data are unavailable; we therefore assumed that mean concentrations in destination provinces reflect combined inputs from all supplying regions and that detection is perfect once sampled, without explicitly modeling analytical sensitivity or method-specific performance. Third, the optimization model is calibrated to a fixed national sampling budget and does not account for potential future policy changes, such as dedicated residue monitoring programs or advanced traceability technologies, which could alter optimal allocation patterns. In addition, we did not explicitly incorporate broader drivers that may influence residue occurrence and interprovincial transfer, including socioeconomic factors (e.g., transportation routes, handling practices, and market demand), climate variability, and heterogeneity in policy enforcement and regulatory capacity; logistics configurations and cold-chain practices may amplify or mitigate contamination risks, while climate change could destabilize supply chains and affect residue management.

## Resource availability

### Lead contact

Requests for further information and resources should be directed to and will be fulfilled by the lead contact, Zhengcong Wang (zhengcong_wang@mail.hzau.edu.cn).

### Materials availability

This study did not generate new unique reagents.

### Data and code availability

The data that support the findings of this study is available in supplemental information, and the optimization codes are available from Github repository (See key resources table). Any additional information required to reanalyze the data reported in this article is available from the lead contact upon reasonable request.

## Acknowledgments

The authors acknowledge the funding provided by 10.13039/501100001809National Natural Science Foundation of China (72303209 and 72403094) and 10.13039/501100002858China Postdoctoral Science Foundation grant (GZB20230644). We kindly thank Dr. Pan He (Cardiff University) for her valuable input to this study.

## Author contributions

Conceptualization: C.J. and Z.W.; methodology: C.J. and Z.W.; investigation: C.J. and Z.W.; visualization: C.J., Z.W., and S.F.; supervision: C.J., Z.W., and W.Z.; writing – original draft: C.J. and Z.W.; writing – review and editing: C.J. and Z.W.

## Declaration of interests

The authors declare no competing interests.

## STAR★Methods

### Key resources table

**Table:** 

REAGENT or RESOURCE	SOURCE	IDENTIFIER
**Software and algorithms**		

Python (version 3.12.4)	Python Software Foundation	http://www.python.org/
LINGO (version 18.0 x64)	LINDO Systems Inc	https://www.lindo.com/

**Deposited data**		

Code availability	Github	https://github.com/puhmli/AQUACTIC-ANTIBIOTIC
Aquatic AR/consumption/trade data	Github	https://github.com/puhmli/AQUACTIC-ANTIBIOTIC

### Method details

We developed a framework to estimate the risk of various AR embedded within China’s aquatic supply chains and to quantify how food safety monitoring influences these risks, leveraging diverse datasets and interdisciplinary methods. The overall framework of our methods is summarized in Figure 2. Our approach followed four main tasks. First, we estimated the contamination proportion and concentration of AR from all aquatic products production regions to respective consumption regions. Second, we calculated the potential food loss due to AR contamination along the aquatic supply chains. Third, we quantified the health risk of human exposure to AR through aquatic products in China, and built the footprints of the health risk transferring from production to consumption regions. Fourth, we developed an optimal food safety monitoring effectiveness model to identify the contaminated aquatic products, and the impacts of the optimal food safety monitoring scheme on food loss and health risk were estimated and compared with the impacts of the currently implemented food safety monitoring. All model parameter values are provided in the Supplementary Data.

#### Data

##### Aquatic AR data

We construct a comprehensive dataset on aquatic AR in China based on the food safety sampling and testing information disclosed by 247 organizations of the State Administration for Market Regulation (SAMR)[https://www.samr.gov.cn/zw/zfxxgk/fdzdgknr/index.html]. This dataset includes data from 11 million food safety tests conducted by SAMR organizations. As stipulated by the China Food Safety Laws, these organizations are mandated to conduct randomized sampling across all segments of the Chinese food supply chain in all the 287 prefecture cities in mainland China and to publicly disseminate the outcomes of these tests. The analytical testing methods applied were by the requisite standards for AR detection. We construct the dataset in the following steps. First, we located all official websites hosting food safety test outcomes. Second, we retrieved all relevant documents, including PDFs, Microsoft Word/Excel files, and HTML tables, from each identified website. Third, we employed various algorithms to extract tabular data from the acquired files, ensuring a comprehensive collection of testing records. Finally, we purged files unrelated to aquatic AR testing, such as the records of non-aquatic items and legal documents pertaining to court cases. We obtained a dataset containing 300,000 records of aquatic safety sampling and testing from the 31 provinces in mainland China from 31 January 2015 to 31 December 2022. Each record contains the following information: the name and category of the aquatic product tested, the time and location of the test, and the pass/fail assessment of the test. For tests assessed as “failed”, the records also contain the name of each exceeded antibiotic (the number of exceeded antibiotics could be more than one), the level of each exceeded antibiotic, and the standard of the assessment. Among approximate 300,000 records, abound 65,000 are for failed samples, and most of them were contaminated by AR. Our main analysis uses failed samples for the estimation of concentration and contamination proportion.

##### Aquatic consumption data

The yearly average consumption data of aquatic products for each province was obtained from the China National Bureau of Statistics (CNBS). To specifically focus on the regulatory challenges within China’s domestic supply chain and governance of local antibiotic residue, this study primarily analyzes the circulation of domestically produced aquatic produced aquatic products. Therefore, imported aquatic products, which are subject to different customs inspection protocols were not included in this analysis.

##### Aquatic domestic trade data

The aquatic product domestic trade data in 34 provinces of China from 2015 to 2022 are extracted from the China Fisheries Statistical Yearbook (2016–2023). These data serve as the basis for simulating inter-provincial trade flows.

#### Food safety contamination embodied along aquatic supply chains

We established detailed food safety contamination flows to explicitly characterize the footprints of different contaminants through the aquatic supply chains from the production regions to consumption regions across 31 provinces of mainland China. The food safety monitoring data for fresh aquatic and their products were obtained from the Administration for Market Regulation (AMR), which covers the name and category of the aquatic product tested, the time and location of the test, and the pass/fail assessment of the test. For tests assessed as “failed”, the records also contain the name of each exceeded hazard (e.g., antibiotic, heavy metal, microbes, and so forth, the level of each exceeded hazard, and the standard of the assessment. These historical monitoring records were from the 31 provinces in mainland China from 31 January 2015 to 31 December 2022 (See data section). Equations 1 and 2 were used to estimate the contamination probability of individual antibiotic residues in aquatic products along the supply chain, from production to consumption regions, based on conditional probability. In addition, these equations were applied to calculate the average concentrations of AR in aquatic products. We assumed that the conditional probability of AR occurring in aquatic products from production regions to consumption regions represents the potential probability of contamination transmission along the aquatic supply chain. We also assumed that the mean concentrations measured in each consumption region reflect the aggregated contributions from all supplying production provinces, given the lack of origin-specific concentration data.

(Equation 1) ProbCi,j,k=(NSCi,jNSTi,j)×(NSCi,j,k∑h=1h=HNSCi,j,h)

Where, *ProbC*_*i*,*j*,*k*_ is the contamination probability of certain AR in aquatic products over all aquatic products in the consumption region j from production region i; *NSC*_*i*,*j*_ is the number of samples contaminated by different kinds of hazards; *NST*_*i*,*j*_ is the total number of samples from production region *i* to consumption region *j* (both contaminated and non-contaminated samples); NSC_i,j,h_ is the number of samples contaminated by hazard *h* at consumption region *j* from production region i; *H* ≥ *k*.

(Equation 2) CLj,k=∑g=1g=GCLi,k,g∑i=1i=INSCi,j,k

Where, *CL*_*j*,*k*_ is the average concentration level of antibiotic *k* in aquatic products at the consumption region *j*. *CL*_*i*,*k*,*g*_ is the concentration level of antibiotic *k* in the certain (g) contaminated aquatic product sample.

#### Estimation of food losses along aquatic supply chains

(Equation 3), (Equation 4), (Equation 5) were used to estimate food losses by linking contamination rates with trade volumes.

(Equation 3) $${TV}_{j,y}={PV}_{j,y}-{EV}_{j,y}+{IV}_{j,y}$$

Where, *TV*_*j*,*y*_ is the trade volume of aquatic products without AR at consumption province *j* at the year of *y*; *PV*_*j*,*y*_ is the production volume of aquatic products at consumption province *j* at the year of *y*; *EV*_*j*,*y*_ is the exported volume of aquatic products from consumption province *j* at the year of *y*; *IV*_*j*,*y*_ is the imported volume of aquatic products to consumption province *j* at the year of *y*; the above aquatic domestic trade data in 31 provinces of China from 2015 to 2022 are extracted from the China Fisheries Statistical Yearbook(CFSY) (Supplementary data).

(Equation 4) $${CTV}_{j,y}={TV}_{j,y}\times \frac{\sum_{i=1}^{i=I}{NSC}_{i,j}}{\sum_{i=1}^{i=I}{NST}_{i,j}}$$

Where, *CTV*_*j*, *y*_ are the losses of trade volume (food losses) due to all contamination in the year of *y*; $\sum_{i=1}^{i=I}{NSC}_{i,j}$ is the number of samples contaminated by different hazards at consumption region *j*; $\sum_{i=1}^{i=I}{NST}_{i,j}$ is the total number of samples (both contaminated and non-contaminated samples) at consumption region *j*.

(Equation 5) $${CTV}_{j,y,k}={TV}_{j,y}\times \frac{\sum_{i=1}^{i=I}{NSC}_{i,j,k}}{\sum_{i=1}^{i=I}{NST}_{i,j}}$$

Where, *CTV*_*j*,*y*,*k*_ are the losses of trade volume (food losses) due to antibiotic residue *k* in the year *y*; $\sum_{i=1}^{i=I}{NSC}_{i,j,k}$ is the number of samples contaminated by antibiotic k in consumption region *j*.

#### Estimation of health risk along the aquatic supply chain

The health risk associated with consuming contaminated aquatic products was quantified using the Acceptable Daily Intake (ADI) of AR present in the aquatic products, expressed in g/kg/day (micrograms per kilogram per day). The ADI represents the maximum amount of a chemical or drug that can be ingested daily through food without posing a significant health risk to consumers (See Table S3). We calculated the Estimated Daily Intake (EDI) for adults in monitoring regions of China, and then determined the Hazard Quotient (HQ) for each antibiotic by comparing the EDI to the ADI. The HQ is a measure of potential health risk from a specific AR in a given monitoring region. Furthermore, the cumulative health risks from exposure to multiple AR in aquatic products in a particular monitoring region were defined as the Hazard Index (HI). In this assessment, an HQ or HI value of 1 or higher was considered indicative of a potential health risk. By integrating these HQ and HI values with the posterior probabilities of antibiotic contamination in aquatic products, we estimated the risk levels associated with aquatic products from different production regions. We also referred to the study by (Wang et al., 2022; Wang et al., 2024), thereby deriving the HI for provinces responsible for monitoring aquatic products. (Equation 6), (Equation 7), (Equation 8), (Equation 9) were applied to estimate the cumulative health risks associated with dietary exposure to AR in aquatic products by comparing the EDI with the ADI and integrating these values with the corresponding contamination rates.

(Equation 6) $${EDI}_{k,j}={CL}_{k,j}\times {DI}_{j}/BW$$

Where, *EDI*_*k*,*j*_ is the estimated daily intake (μg/kg/person) of antibiotic *k* at the monitoring region *j*; *CL*_*k*,*j*_ is the concentration level (μg/g) of antibiotic *k* at the monitoring region *j*; *DI*_*j*_ is the daily intake of aquatic products (kg/day/person) at monitoring *j*; *BW* is the body weight (kg/person) for adults in China.

(Equation 7) $${HQ}_{k,j}={EDI}_{k,j}/{ADI}_{k}$$

Where, *HQ*_*k*,*j*_ is the potential health risk by consumption of aquatic products contaminated by a specific antibiotic *k* in a given monitoring region *j*; *ADI*_*k*_ is the maximum amount of the antibiotic *k* that can be ingested daily through food without posing a significant health risk to consumers.

(Equation 8) $${CHQ}_{k,i,j}={ProbC}_{i,j,k}\times {HQ}_{k,j}$$

Where, *CHQ*_*k*,*i*,*j*_ is the corrected potential health risk by consumption of aquatic products contaminated by a specific antibiotic *k* in a given monitoring region *j*, considering the conditional probability *ProbC*_*i*,*j*,*k*_ that aquatic products are contaminated by individual antibiotics *k* among all contaminated aquatic product samples from the production region *i* at a given monitoring region *j*.

(Equation 9) $${HI}_{j}=\sum_{i=1}^{i=I}\sum_{k=1}^{k=K}{CHQ}_{i,j,k}$$

Where *HI*_*j*_ is the cumulative health risk from exposure to multiple antibiotics in aquatic products in the consumption region *j*.

#### Impacts of food safety monitoring along the aquatic supply chains

To inform the policy making in aquatic antibiotic risk control, we further assessed the potential impacts of food safety monitoring schemes on mitigating risks related to AR transferring from production regions to consumption regions along the aquatic supply chains. First, we conducted an evaluation of the effectiveness of food safety monitoring, which was defined as the joint probability of identifying the contaminated aquatic products from all production regions at a certain consumption (monitoring) region. We optimized monitoring resources under food safety authority requirements aiming at maximizing the probability of identifying contaminated aquatic products (with contaminants higher than legal limits) from all production regions at each consumption region. Resource allocation in food safety monitoring is key to protecting public health from exposure to hazards in food products. These problems aim to minimize monitoring costs or maximize effectiveness given some constraints and could be formulated by mathematical programming.^72^^,^^86^^,^^87^ In this study, we will use the Integer Programming (IP) to simulate the second monitoring scenario (Figure 2). Then the impacts of optimal food safety monitoring and the monitoring scheme currently implemented on the risk of food loss and health would be quantified and compared for each consumption region.

##### The food safety monitoring effectiveness model and impacts evaluation model

The effectiveness of food safety interventions has been studied from various perspectives, including economic, public health, and social welfare.^87^^,^^88^^,^^89^ In our study, we define the effectiveness of a monitoring scheme as the probability of identifying contaminated products from all production sources. (Equation 10), (Equation 11), (Equation 12), (Equation 13), (Equation 14) were used to evaluate the impacts of different monitoring strategies on food loss and health risk by linking the joint probability of detecting aquatic products contaminated with AR under each monitoring scheme. We assumed that once contaminated products were sampled under a given monitoring scheme, the analytical method (HPLC–GC) would correctly identify the contamination, without explicitly modeling the method’s sensitivity.

(Equation 10) $${g\left(x\right)}_{j,s,ant}=\prod_{i=1}^{i=I}{\left(1-\left(\begin{array}{c} x_{i,j,s}\\ 0 \end{array}\right)a_{i,j,ant}^{0}{\left(1-a_{i,j,ant}\right)}^{x_{i,j,s}}\right)}_{i}$$

Where, *g*(*x*)_*j*,*s*,*ant*_ is the probability of identifying the aquatic products contaminated by AR from all geographical sources at the same time under the monitoring scenario *s*; $\left(\begin{array}{c} x_{i,j,s}\\ 0 \end{array}\right)a_{i,j,ant}^{0}{\left(1-a_{i,j,ant}\right)}^{x_{i,j,s}}$ is following the binomial distribution and represents the probability of non-antibiotic-contaminated products that could be identified at the consumption region *i*,under scenario *s*; *x*_*i*,*j*,*s*_ is the number of samples collected and tested at the consumption province *j* for aquatic products from production province *i* under scenario *s*; *a*_*i*,*j*,*ant*_ is the contamination probability of aquatic products due to AR from production province *i* at consumption province *j*.

(Equation 11) $${STV}_{j,s,ant}=\frac{\sum_{y=1}^{y=Y}{CTV}_{j,y,k}}{Y}\times \left(1-a_{j,ant}\times {g\left(x\right)}_{j,s,ant}\right)$$

Where, *STV*_*j*,*s*,*ant*_ is the saved food loss due to AR along the aquatic products by monitoring scenario *s* at consumption region *j*; *Y* is the number of years involved in the historical monitoring records; *a*_*j*,*ant*_ is the contamination probability of aquatic products due to AR at consumption province *j*.

(Equation 12) $$\Delta {STV}_{j,ant}={STV}_{j,o,ant}-{STV}_{j,2022,ant}$$

Where, Δ*STV*_*j*,*ant*_ is the comparison between the impacts of these two monitoring scenarios on the risk of food loss due to AR along the aquatic products at the consumption region *j*; *STV*_*j*,*o*,*ant*_ is the saved food loss due to AR along the aquatic products by the optimal monitoring scenario at the consumption region *j*; *STV*_*j*,2022,*ant*_ is the saved food loss due to antibiotic residues along the aquatic products by the monitoring scenario in 2022 at the consumption region *j*.

(Equation 13) $${SHI}_{j,s}=\sum_{i=1}^{i=I}{\left(1-\left(\begin{array}{c} x_{i,j,s}\\ 0 \end{array}\right)a_{i,j}^{0}{\left(1-a_{i,j}\right)}^{x_{i,j,s}}\right)}_{i}\times {HI}_{i,j}$$

Where, *SHI*_*j*,*s*_ is the saved health risk due to antibiotic residues under the monitoring scenario *s* at consumption region *j*; ${\left(1-\left(\begin{array}{c} x_{i,j,s}\\ 0 \end{array}\right)a_{i,j}^{0}{\left(1-a_{i,j}\right)}^{x_{i,j,s}}\right)}_{i}$ is the probability of the monitoring scheme at consumption province *j* could identify aquatic products contaminated by antibiotic residues from province *i* under scenario *s*; *HI*_*i*,*j*_ is the cumulative health risks from exposure to multiple antibiotics in aquatic products from the production region *i* at the consumption region *j*.

(Equation 14) $$\Delta {HI}_{j}={SHI}_{j,o}-{SHI}_{j,2022}$$

Where, Δ*HI*_*j*_ is the comparison between the impacts of these two monitoring scenarios on the health risk due to AR along the aquatic products in the consumption region *j*; *SHI*_*j*,*o*_ is the saved health risk due to AR along the aquatic products by the optimal monitoring scenario at the consumption region *j*; *SHI*_*j*,2022_ is the saved health risk due to AR along the aquatic products by the monitoring scenario in 2022 in the consumption region *j*.

##### Optimal allocation of food safety monitoring resources

Optimization of food safety monitoring resources from a cost-effectiveness perspective is a core challenge in food safety risk management. Such problems, often centered on minimizing monitoring costs or maximizing effectiveness within given constraints, can be approached through mathematical programming.^86^^,^^87^^,^^90^ In this study, we apply integer programming (IP) with historical monitoring data to maximize the probability of identifying contaminated aquatic products, defined as those exceeding legal limits, while adhering to current authority requirements. Previous research demonstrates the potential of maximizing sampling and testing effectiveness in settings with limited laboratory, enterprise, and government capacities,^86^^,^^87^^,^^91^ underscoring the need for a balanced, cross-sectoral approach to managing high-risk contaminants. Building on these insights, our framework systematically considers how food safety risks transfer through the supply chain and across regions, which is crucial for refining resource allocation in seafood supply chain risk regulation and traceability. (Equation 15), (Equation 16), (Equation 17), (Equation 18), (Equation 19), (Equation 20), (Equation 21) were applied to optimize the monitoring resources allocation to identify contaminated aquatic products with subject to constraints on resource availability, trade volumes, and health risks from AR.

(Equation 15) $$\max_{x_{i,j}}{h\left(x\right)}_{j}=\prod_{i=1}^{i=I}{\left(1-\left(\begin{array}{c} x_{i,j}\\ 0 \end{array}\right)a_{i,j}^{0}{\left(1-a_{i,j}\right)}^{x_{i,j}}\right)}_{i}$$

s.t.

(Equation 16) $$\sum_{i=1}^{i=I}x_{i,j}\leq {NS}_{j,2022}$$

(Equation 17) $${TV}_{j}=\left({PV}_{j}-{EV}_{j}+{IV}_{j}\right)\times \left(1-a_{j}\times {Prob}_{i,j}\right)$$

(Equation 18) $${TV}_{j}\geq {TV}_{j,mean}$$

(Equation 19) $$\Delta {HI}_{j}=\sum_{i=1}^{i=I}{\left(1-\left(\begin{array}{c} x_{i,j}\\ 0 \end{array}\right)a_{i,j,ant}^{0}{\left(1-a_{i,j,ant}\right)}^{x_{i,j}}\right)}_{i}\times {HI}_{i,j}$$

(Equation 20) $${HI}_{j}-\Delta {HI}_{j}\leq Standard\_value$$

(Equation 21) $$x_{i,j}\geq 0;x_{i,j}\in Z$$

Where, *h*(*x*)_*j*_ is the effectiveness of the monitoring scheme at province *j*, which is defined as the probability of identifying the contaminated aquatic products from all geographical sources at the same time; $\left(1-\left(\begin{array}{c} x_{i,j}\\ 0 \end{array}\right)a_{i,j}^{0}{\left(1-a_{i,j}\right)}^{x_{i,j}}\right)$ is the probability of the monitoring scheme at consumption province *j* could identify contaminated aquatic products from province *i*; *x*_*i*,*j*_ is the number of samples collected and tested at consumption province *j* for aquatic products from production province *i*; *a*_*i*,*j*_ is the contamination probability of aquatic products from production province *i* at consumption province *j*; *a*_*i*,*j*,*ant*_ is the contamination probability of aquatic products due to AR from production province *i* at consumption province *j*; *NS*_*j*,2022_ is the total number of samples collected and tested at consumption province *j* in 2022; *Prob*_*i*,*j*_ is the probability that the monitoring scheme at consumption province *j* in 2022 could identify the contaminated aquatic products from all geographical sources; *a*_*i*,*j*_ is the contamination probability of aquatic products at consumption province *j*; Standard value is the threshold to reflect the health risk, and is defined as 1; *TV*_*j*_ is the trade volume of aquatic products without AR at consumption province *j*; *PV*_*j*_ is the production volume of aquatic products at consumption province *j*; *EV*_*j*_ is the exported volume of aquatic products from consumption province *j*; *IV*_*j*_ is the imported volume of aquatic products to consumption province *j*; *TV*_*j*,*mean*_ is the average historical trade volume of aquatic products without AR at consumption province *j*; *HI*_*j*_ is the cumulative health risk from exposure to multiple antibiotics in aquatic products at the monitoring *j*; Δ*HI*_*j*_ is the reduced cumulative health risk from exposure to multiple antibiotics in aquatic products at the monitoring *j* by monitoring scheme.

#### Sensitivity analysis

To assess the robustness of the model outputs to parameter uncertainty, we conducted one-at-a-time deterministic sensitivity analyses. Mean AR concentrations were varied by ±20%, consistent with the typical analytical uncertainty and precision reported for HPLC-GC (High-Performance Liquid Chromatography-Gas Chromatography) residue. Contamination probability was adjusted by ±10% to represent plausible sampling variability and inter-annual fluctuations in failure proportions reflected in historical monitoring data and also the sensitivity of HPLC-GC (98%) was included. These ranges align with the magnitude of analytical method precision and sampling uncertainty reported in residue method validation and uncertainty assessment guidelines.^92^ Under each perturbed scenario, we recalculated health risks, food loss estimates, and the effectiveness of alternative monitoring strategies, and compared these results with the baseline to evaluate the stability of the model’s quantitative conclusions.

### Quantification and statistical analysis

The contamination estimation and assessment of food loss and health risk processes were developed using Python (version 3.12.4) and Microsoft Excel 2021(Microsoft Corporation, Redmond, WA, USA). The optimization model and mathematical programming were developed using LINGO (version 18.0 x64). All the data and codes were presented in https://github.com/puhmli/AQUACTIC-ANTIBIOTIC.
