# Supplementary material for: Hidden middle governance reduces antibiotic residue health risks and food loss in aquatic supply chains
Source: iScience. 2026 Feb 20;29(3):115097. doi: 10.1016/j.isci.2026.115097 (PMC12989963; doi:10.1016/j.isci.2026.115097)
Supplement: Document S1. Figures S1–S11 and Tables S1–S3 [file mmc1.pdf]

iScience, Volume *29*

Supplemental information

Hidden middle governance reduces antibiotic residue health risks and food loss in aquatic supply chains

Cangyu Jin, Zhengcong Wang, Weihua Zhou, and Siwei Fu

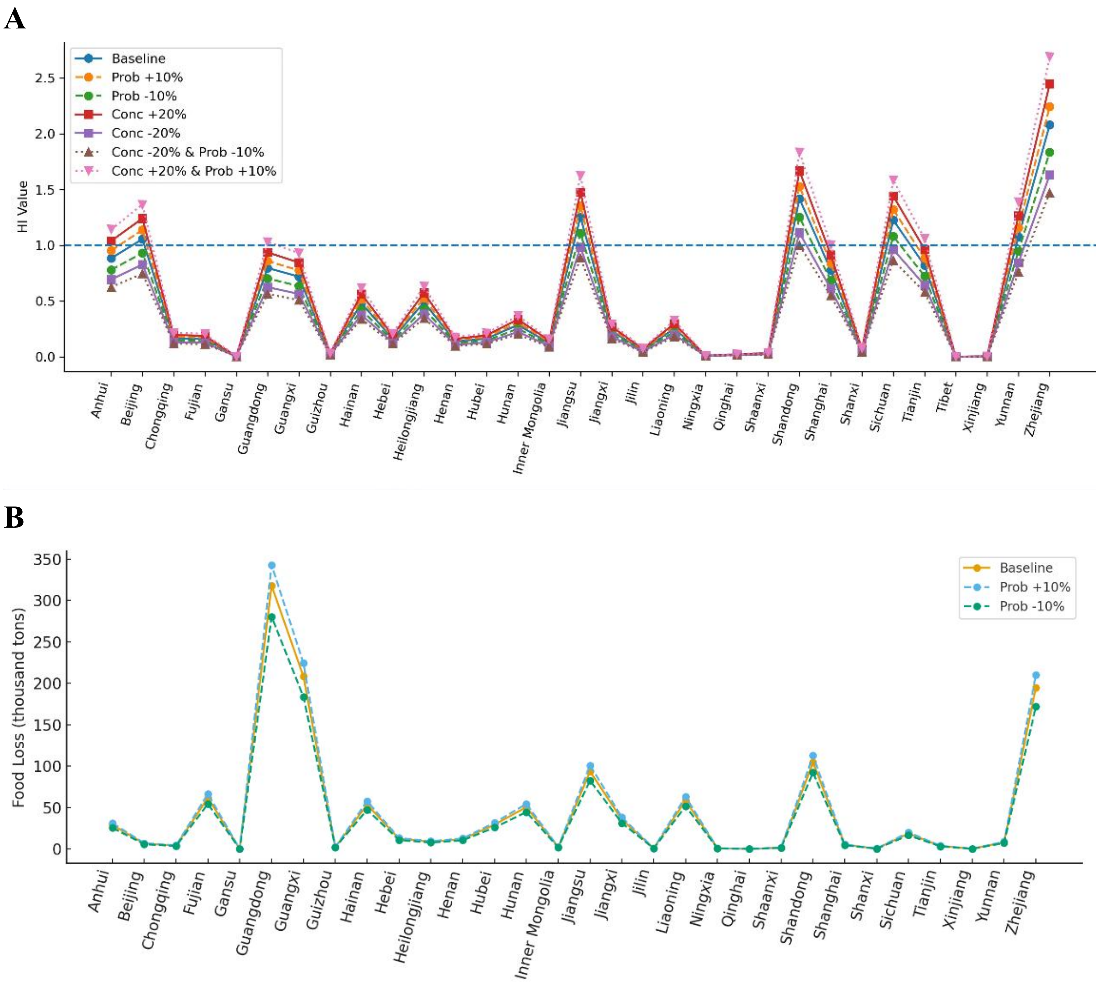

Figure S1. Sensitivity analysis of (A) health risk, expressed as the hazard index (HI), and (B) food loss, accounting for uncertainty in contamination probability (±10%), concentration (±20%), and a detection method sensitivity of 98% (HPLC-GC), Related to Figure 5.

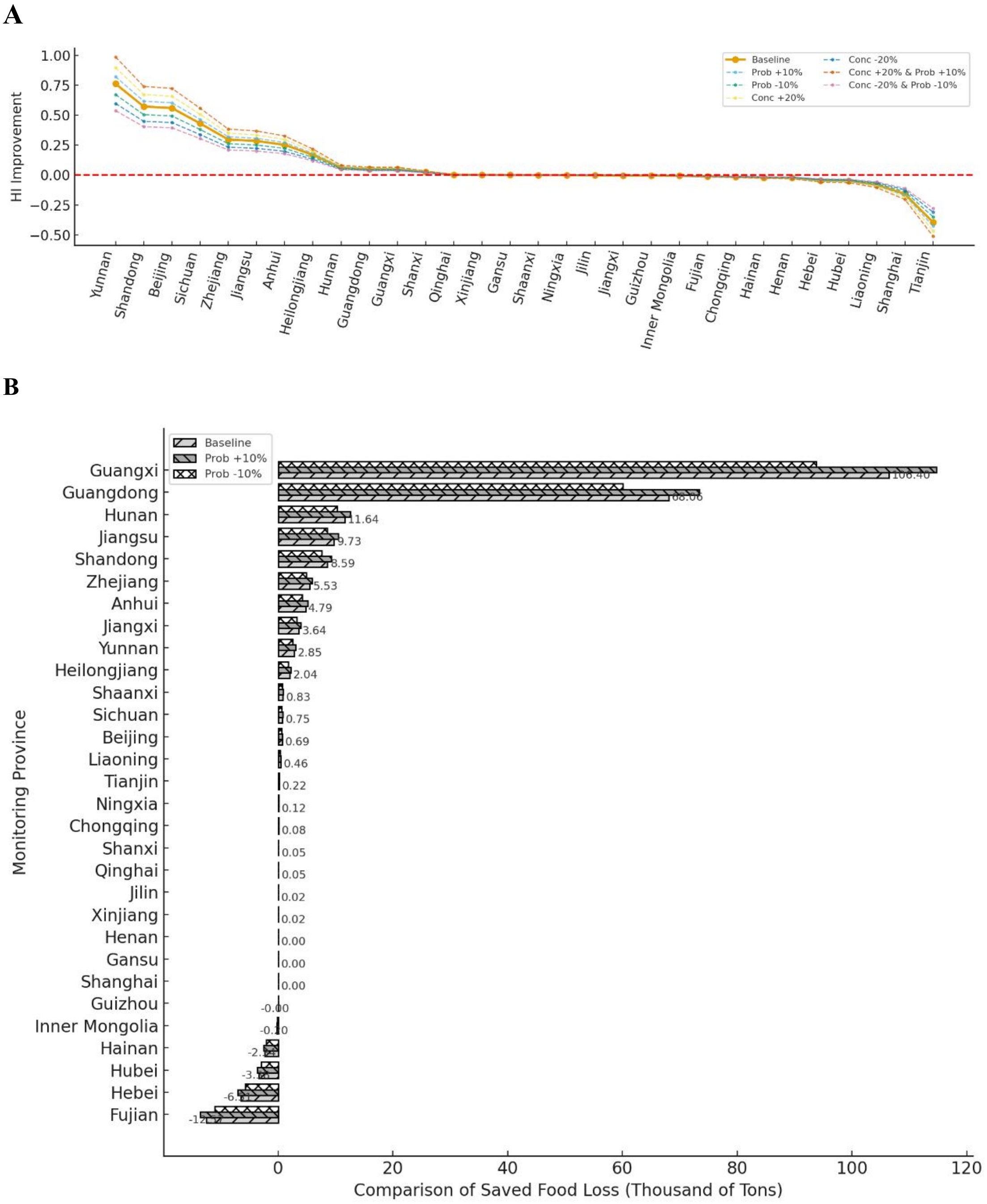

Figure S2. Sensitivity analysis of the impacts of optimal versus conventional monitoring on (A) health risk, expressed as the hazard index (HI), and (B) avoided food loss, incorporating uncertainty in contamination probability (±10%), concentration (±20%), and a detection method sensitivity of 98% (HPLC–GC), Related to Figure 7.

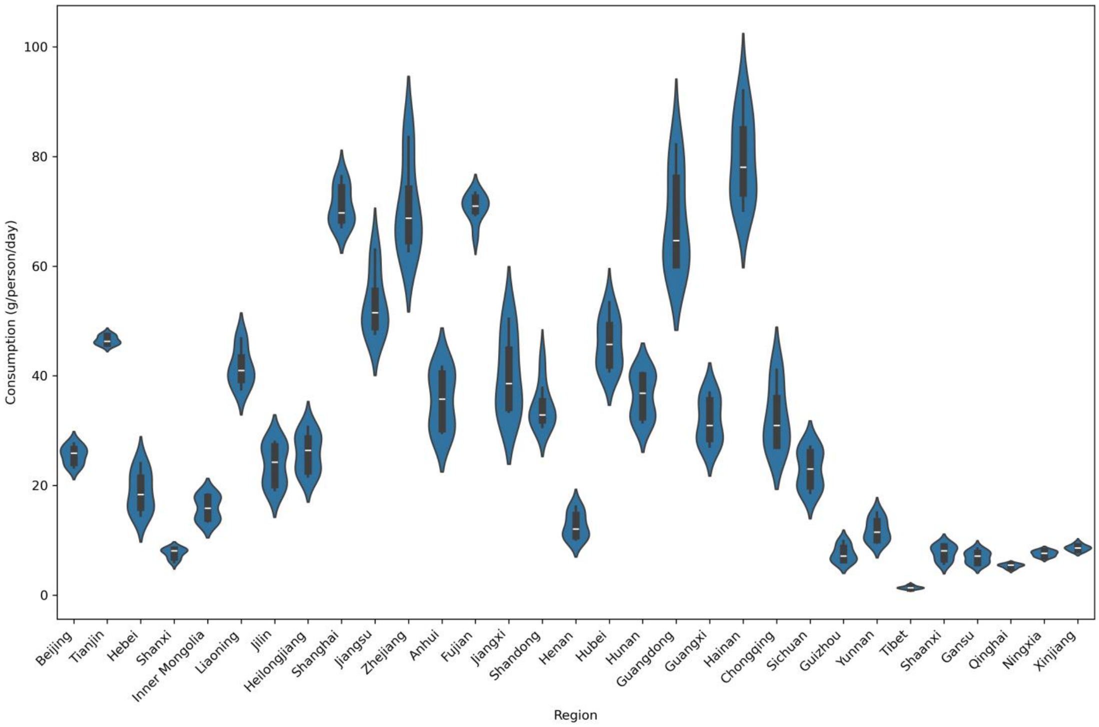

Figure S3. Consumption distribution of aquatic products at each region of China (2015-2022), Related to Figure 5.

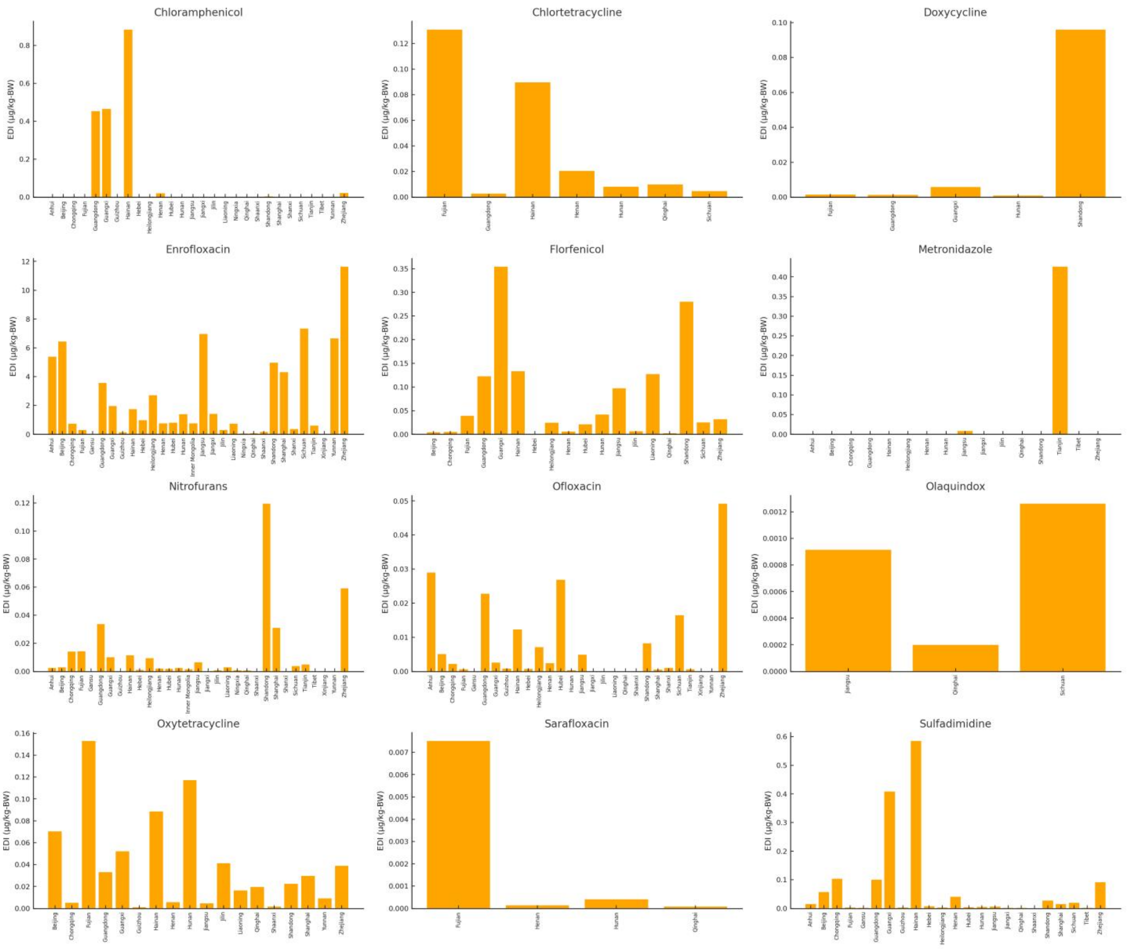

Figure S4. The estimated daily intake (EDI) of different antibiotics through consumption of aquatic products at each monitoring province, Related to Figure 5.

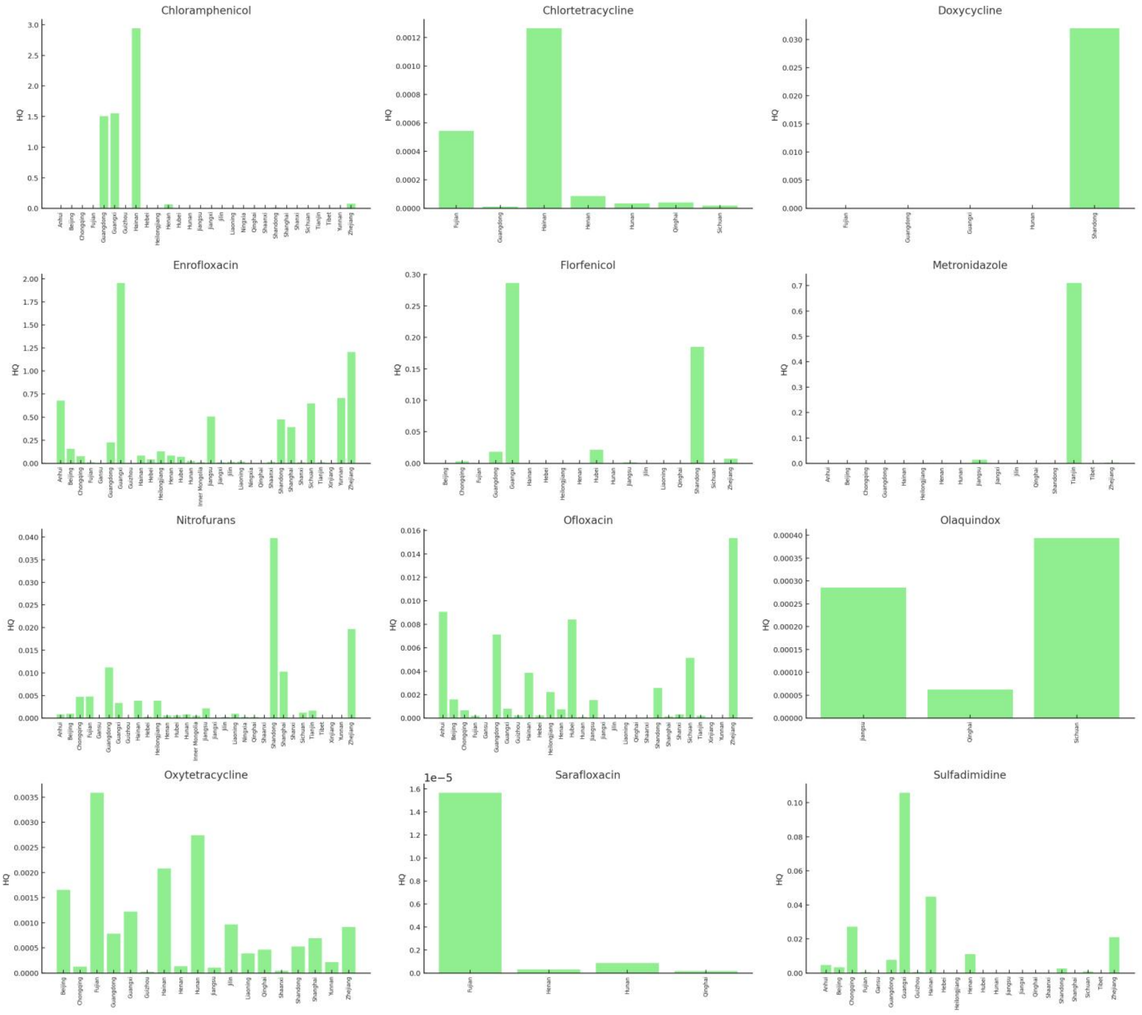

Figure S5. The hazard index (HQ) of different antibiotics through consumption of aquatic products at each monitoring province, Related to Figure 5.

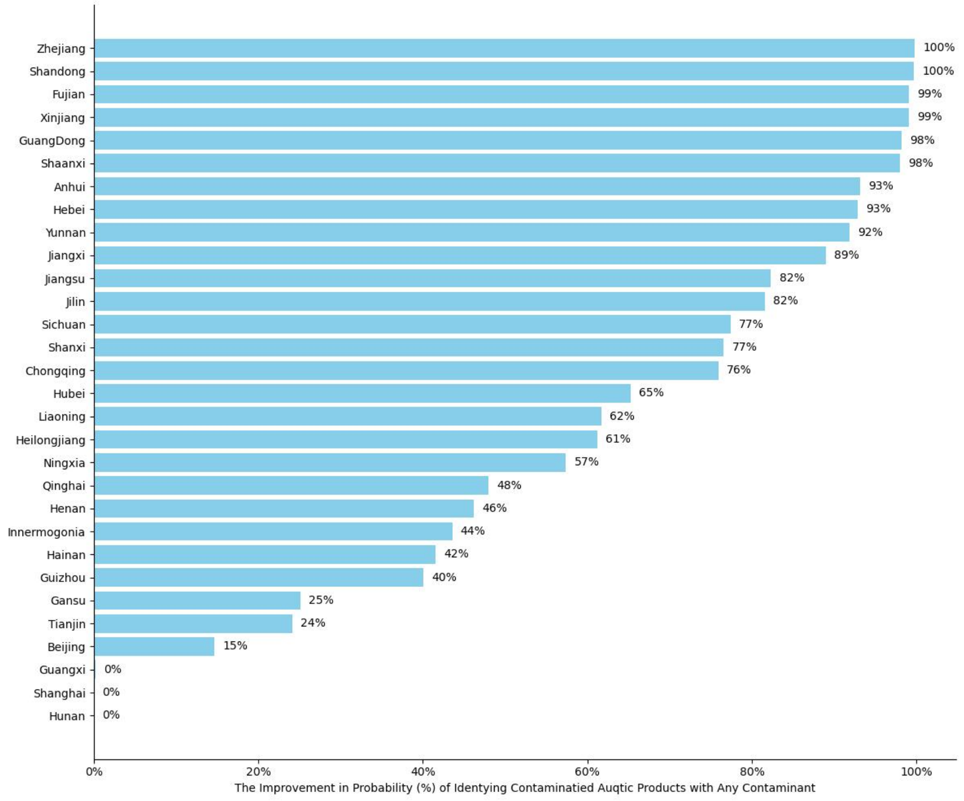

Figure S6. The improvement probability of identifying contaminated aquatic products with any contaminant between optimal monitoring and baseline monitoring, Related to Figure 7.

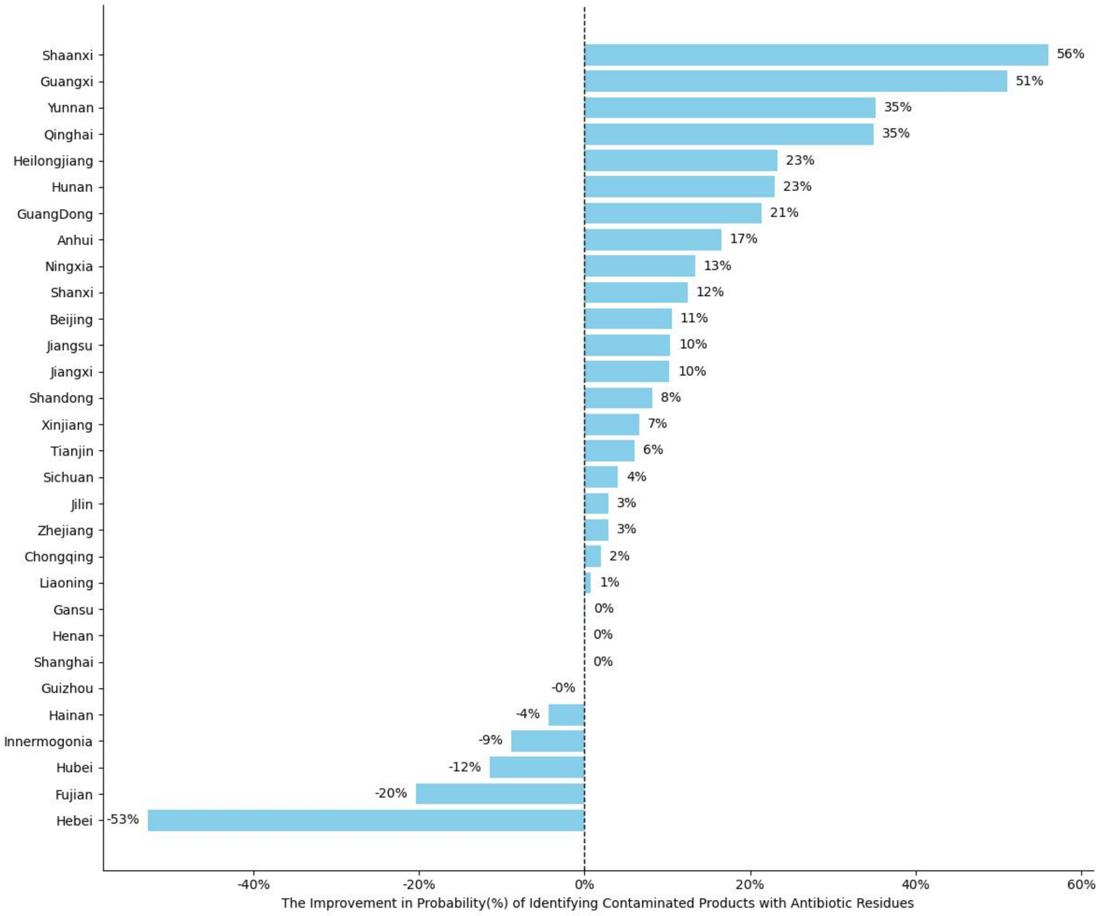

Figure S7. The improvements of probability contaminated with antibiotic residue between baseline monitoring and optimal monitoring, Related to Figure 7.

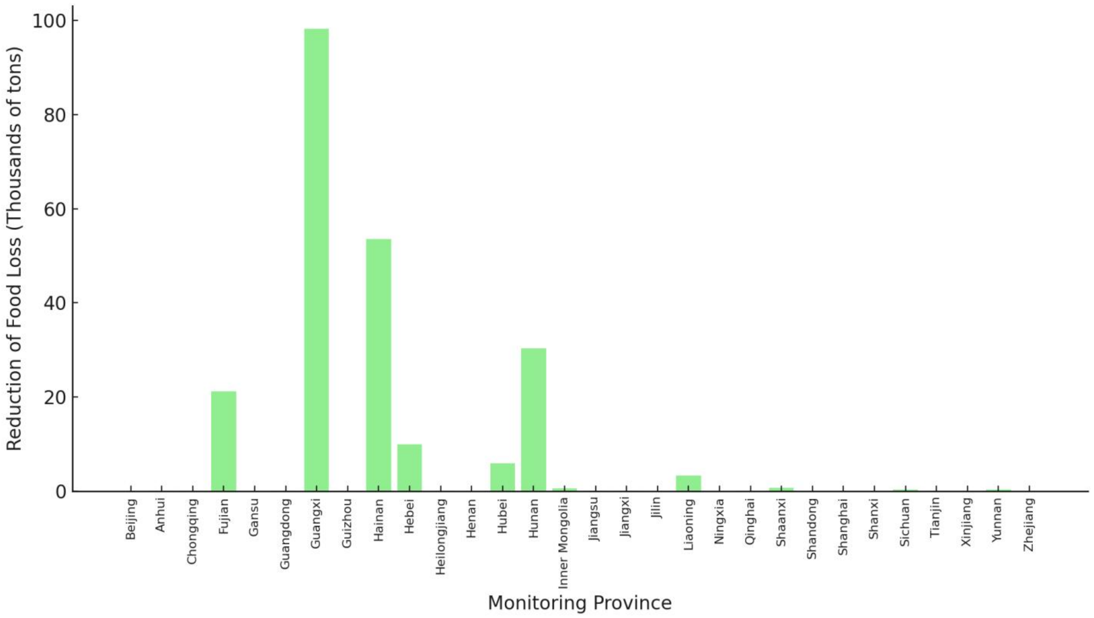

Figure S8. Reduction of food loss due to antibiotic residues by baseline monitoring at each province.

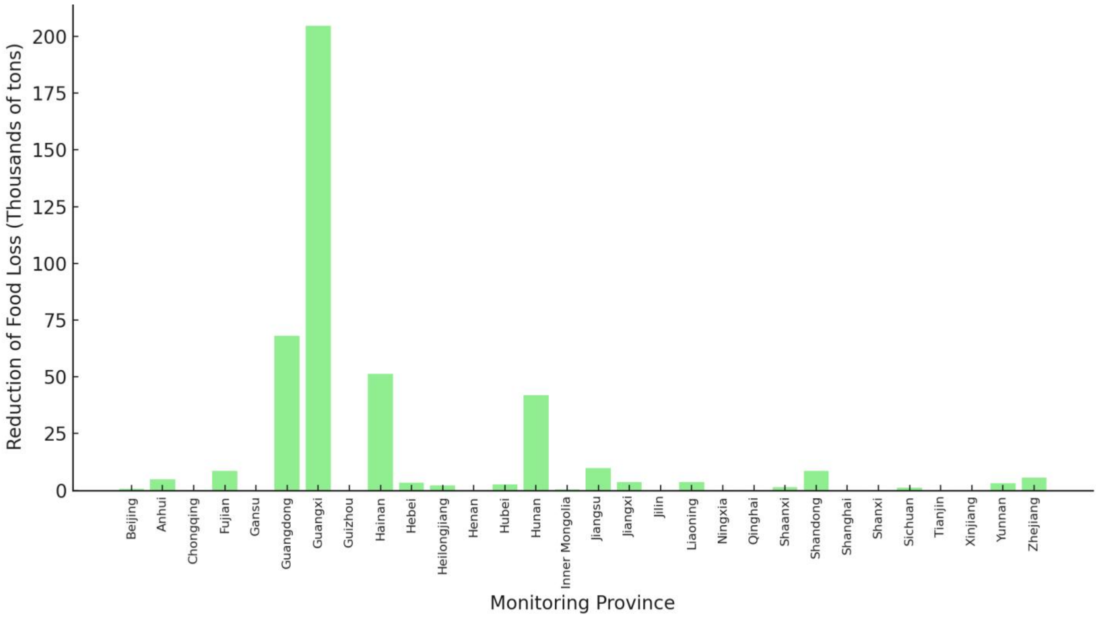

Figure S9. Reduction of food loss due to antibiotic residues by optimal monitoring at each province, Related to Figure 7.

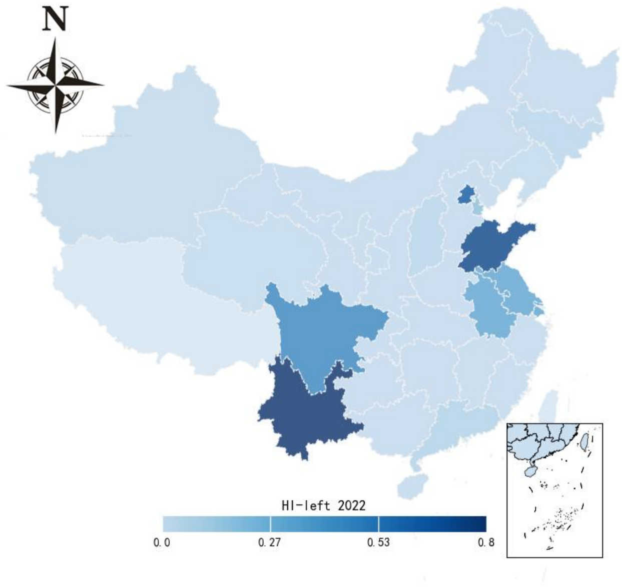

Figure S10. HI values in 2022, with darker blue colors indicating higher antibiotic residue HI levels in the corresponding province, Related to Figure 5.

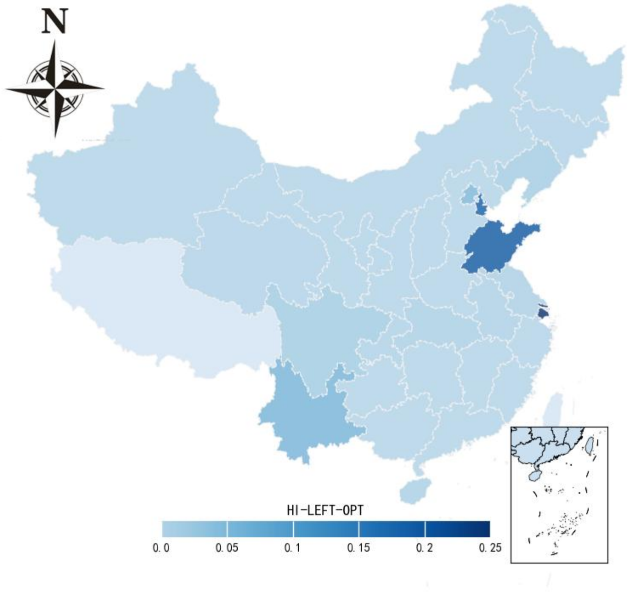

Figure S11. Optimized HI values, with darker blue colors indicating higher antibiotic residue HI levels in the corresponding province, Related to Figure 7.

Tables S1. Acceptable daily intake, regulation limits and limit of detection for antibiotics, Related to Figure 3.

Antibiotics	Acceptable Daily Intake (μg/kg-BW/day)[a]	Maximum Level (μg/kg)[b]	Limit of Detection (μg/kg)[c]
Enrofloxacin	6.2	100	0.1
Nitrofurans	0.4	0	1
Quinolones	30	0	1
Chloramphenicol	–	0	0.16
Sulfadimidine	25	100	0.43
Oxytetracycline	15	200	15
Florfenicol	1	100	0.02
Chlortetracycline	30	200	0.5
Sarafloxacin	0.33	30	0.5
Metronidazole	0.6	0	0.46
Olaquindox	7	0	0.5
Doxycycline	3	100	0.49
Ofloxacin	3.2	2	0.43

a. Source: WHO, 1998; EPA, 2003.

b. Source: GB regulations.

c. Detection limits of HPLC-GC for each antibiotic sourced from relevant studies.

Tables S2. Aquatic category included in study, Related to Figure 1.

Food category
Freshwater Shrimp
Marine Fish
Marine Shrimp
Freshwater Fish
Freshwater Crab
Shellfish
Dried Aquatic Products
Marine Crab
Other Aquatic Products
Cooked Animal Aquatic Products
Raw Animal Aquatic Products
Salted Aquatic Products
Pre-prepared Surimi Products
Other Aquatic Product Preparations

Tables S3. The Source of ADI, Related to Figure 2 and 5.

Name	ADI (ug/kg-BW)	Source of ADI
Enrofloxacin	6.2	European Medicines Agency (EMA): https://www.scirp.org/journal/paperinformation?paperid=63869
Nitrofurans	0.4	EFSA: https://efsa.onlinelibrary.wiley.com/doi/pdf/10.2903/j.efsa.2015.4140
Quinolones	30	https://agriculture.gov.au/sites/default/files/sitecollectiondocuments/aqis/importing/food/notices/fsanz.pdf
Chloramphenicol	–	WHO/FAO and EMA: https://efsa.onlinelibrary.wiley.com/doi/pdf/10.2903/j.efsa.2014.3907
Sulfadimidine	25	WHO: https://apps.who.int/food-additives-contaminants-jecfa-database/Home/Chemical/3194
Oxytetracycline	15	WHO: https://apps.who.int/food-additives-contaminants-jecfa-database/Home/Chemical/3859
Florfenicol	1	Australian Government. Acceptable Daily Intakes for Agricultural and Veterinary Chemicals 2014, Department of Health and Ageing Office of Chemical Safety, Canberra (2014)
Chlortetracycline	30	WHO: https://apps.who.int/food-additives-contaminants-jecfa-database/Home/Chemical/628
Sarafloxacin	0.33	WHO: https://apps.who.int/food-additives-contaminants-jecfa-database/Home/Chemical/2867
Metronidazole	0.6	https://pubchem.ncbi.nlm.nih.gov/compound/Metronidazole#section=Chemical-Co-Occurrences-in-Literature
Olaquindox	7	WHO: https://apps.who.int/food-additives-contaminants-jecfa-database/Home/Chemical/4060
Doxycycline	3	EMA: https://www.ema.europa.eu/en/documents/mrl-report/doxycycline-all-food-producing-species-european-public-maximum-residue-limit-assessment-report-epmar-cvmp_en.pdf
